# A Jack of All Trades: Impact of Glucocorticoids on Cellular Cross-Talk in Osteoimmunology

**DOI:** 10.3389/fimmu.2019.02460

**Published:** 2019-10-17

**Authors:** Mubashir Ahmad, Yasmine Hachemi, Kevin Paxian, Florian Mengele, Mascha Koenen, Jan Tuckermann

**Affiliations:** ^1^Institute of Comparative Molecular Endocrinology (CME), University of Ulm, Ulm, Germany; ^2^Praxisklinik für Orthopädie, Unfall- und Neurochirurgie Prof. Bischoff/ Dr. Spies/ Dr. Mengele, Neu-Ulm, Germany; ^3^Department of Immunobiology, Yale School of Medicine, New Haven, CT, United States

**Keywords:** glucocorticoids, glucocorticoid receptor, osteoporosis, arthritis, inflammation, fracture healing, conditional knockout mice

## Abstract

Glucocorticoids (GCs) are known to have a strong impact on the immune system, metabolism, and bone homeostasis. While these functions have been long investigated separately in immunology, metabolism, or bone biology, the understanding of how GCs regulate the cellular cross-talk between innate immune cells, mesenchymal cells, and other stromal cells has been garnering attention rather recently. Here we review the recent findings of GC action in osteoporosis, inflammatory bone diseases (rheumatoid and osteoarthritis), and bone regeneration during fracture healing. We focus on studies of pre-clinical animal models that enable dissecting the role of GC actions in innate immune cells, stromal cells, and bone cells using conditional and function-selective mutant mice of the GC receptor (GR), or mice with impaired GC signaling. Importantly, GCs do not only directly affect cellular functions, but also influence the cross-talk between mesenchymal and immune cells, contributing to both beneficial and adverse effects of GCs. Given the importance of endogenous GCs as stress hormones and the wide prescription of pharmaceutical GCs, an improved understanding of GC action is decisive for tackling inflammatory bone diseases, osteoporosis, and aging.

## Introduction

Glucocorticoids (GCs) form one major axis of the stress response (1) and are used as immunosuppressive therapeutics in a variety of inflammatory bone diseases (2, 3). Strong impact on innate immune cells, namely macrophages, dendritic cells, and mast cells, contribute to the inhibition of inflammation. On the other hand, GCs are known to cause the most frequent secondary osteoporosis at conditions of high GC exposure. In this process myeloid cells, osteoclasts, and mesenchymal cells and their derivatives, chondrocytes, osteoblasts, and osteocytes are affected. Whereas, the cell-autonomous roles of GCs acting via the nuclear glucocorticoid receptor (GR) had been investigated intensively, the knowledge about the influence of GCs on cross-talk between innate immune cells, mesenchymal cells, and bone cells is scarce. How GCs act on cellular interactions in the osteo-immunological network is currently unraveled and is subject to this review.

## Glucocorticoids (GCs), Stress Hormones and Anti-inflammatory Agents

Two different axes initiate the human physiological reaction to stress. While the activation of sympathetic-adrenal medulla (SAM)-axis starts a short-term stress reactions, long-term stress responses are mediated by the hypothalamus-pituitary-adrenal (HPA)-axis. Stress exposure results in the releases of corticotrophin-releasing hormone (CRH) from the hypothalamus, causing the synthesis of adrenocorticotropic hormone (ACTH) in the anterior pituitary gland, which activates the production of GCs in the adrenal cortex via induction of key enzymes of steroid synthesis (4).

Under long-term stress conditions GC release from the adrenal cortex also results in diverse physiological adaptations. Cortisol activates gluconeogenesis in the liver, decreases pancreatic insulin secretion, and promotes the release of glucagon. Furthermore, blood pressure elevates, the effect of catecholamines is potentiated, and a mild sodium/water-retention induced (5).

Since the first successful treatment of arthritis (6), GCs have been in frequent use and approximately 3% of the elderly population are being treated with GCs (7, 8), to reduce inflammatory symptoms in acute and chronic inflammatory diseases, including rheumatoid and osteoarthritis.

Adverse side effects of GCs on the human body have been observed upon extended treatment with daily prednisolone-doses of 7.5 mg and above. Besides the Cushingoid phenotype and osteoporosis, metabolic side effects as peripheral insulin resistance, type 2 diabetes and dyslipidemia are predominant (1). In addition, atrophy of skin and impact on the central nervous system can occur. To a similar extent, long-term GC treatment affects the cardiovascular system, resulting in hypertension, thrombotic stroke or myocardial infarction (9). These well-known side effects often preclude long-term treatment and cause occasional severe long lasting damage to the patient. Given the strong acute action of GCs to reduce inflammation, however, side effects are accepted to a certain extent in clinical praxis.

At the molecular level, intracellular GC-activity depends on the enzymes 11β-hydroxysteroid dehydrogenase type 1 and 2 (11β-HSD1 and 11β-HSD2). 11β-HSD1 catalyzes the conversion of cortisone into active cortisol, 11β-HSD2 mainly induces the reverse reaction by inactivating cortisol (10). A specific ratio of both isozymes is given in different tissue types, for example 11β-HSD1 being predominant in liver and adipose tissue (11). Molecular actions of GCs are initiated by binding to the mineralocorticoid receptor (MR) and the GC receptor (GR). Due to the wide expression of GR compared to MR and the inactivation of GCs by 11β-HSD2 in MR high expressing tissues, most of the GC effects are mediated by the GR as evident from knockout studies. However, the role of MR in inflammation is becoming more recognized and is reviewed elsewhere (12). The GR belongs to the nuclear receptor superfamily and acts as a ligand-induced transcription factor, resulting in transactivation or transrepression of genes (10). The GR structure is constituted by four domains: the transactivation domain AF1/2 (docking station for co-regulators and regulative enzymes), the DNA-binding-domain, the ligand-binding domain (binding locus for GCs) and the hinge-region (involved in translocation of GR) (10). When located in the cytoplasm GR, is in a state of high affinity to GCs and captured in a complex with immunophilins (FKBP51), heat-shock-proteins (Hsp90) and p23 (13). GC binding leads to an exchange of FKBP51 into FKBP52, resulting in translocation of the protein complex via interaction with the microtubules (10, 13). In case of nuclear transactivation, the GR tends to dimerize and bind to specific motives on target DNA, the GC response element (GRE). The ability of GCs to downregulate genes is mediated in part by GR-binding to negative GREs and consecutive recruitment of corepressors; all leading to deacetylation of histones and decrease of gene transcription [reviewed in (10, 14)]. A “tethering mode” whereby a GR-monomer interacts with DNA-bound inflammatory transcription factors (NF-κB, AP-1, STAT3, IRF3) instead of directly binding to DNA was observed for the repression of genes encoding pro-inflammatory mediators, such as cytokines and matrix metalloproteases (15). This way of cytokine-transrepression eventually leads to immunosuppression. Furthermore, crosstalk exists between DNA-bound GRs and NF-κB or AP-1 bound to transcription-factor binding-sites in the vicinity. However, both mechanisms—transactivation via dimerized GRs and transrepression via tethering of monomeric GR—are obligatory for complete anti-inflammatory GC actions (16). Non-genomic GR-effects can be observed under high-dose GC-application and modulated by GR-interaction with membranes or mitochondria (3).

Short term rise in physiological levels of GCs can stimulate the immune function, whereas immunosuppression resulting from chronic stress, favors infections or tumorigenesis (17). The immunomodulatory actions of GCs are amongst other functions achieved by priming of innate immunity. Under physiological stress conditions macrophage phagocytosis, natural killer-cell activity and cytokine production are increased (17). Furthermore, a wide range of stress-effects on leukocytes is observed: ranging from enhanced proliferation and distribution in the lymphatic system or better endothelial adhesion, to leukocyte margination and transmigration into the inflamed tissue (17). In contrast, chronically elevated GCs levels impair leukocyte proliferation and redistribution and cytokine and prostaglandin synthesis (17).

Accordingly Frank et al. (18) showed that GCs play an important role as an alarmin in neuroinflammatory priming. Stress induced high GC levels result in NLRP3 inflammasome priming, whereby the innate immune system (e.g., microglia) switches into activation mode (18). Frank et al. describe this paradox GC-induced neuroimmune activation under neuroinflammatory conditions to be an adaptive way of preparing against potential neuronal injuries or infections (18).

Thus, GCs via the GR suppress inflammatory reactions, but may also stimulate them, depending on pharmacological conditions. Whereas, for immune suppression several molecular mechanisms of the GR, transactivation of anti-inflammatory acting genes and repression of pro-inflammatory acting genes is required, the mode of action for immune priming remains elusive.

How the different modes of action of the GR impact osteoimmunological cross-talk by influencing bone and immune cells is discussed in this review.

## Glucocorticoid (GC) Action on Bone: Direct Effects and the Modulation of the Crosstalk of Bone Cells

### Cell Autonomous Effects of GCs on Bone Cells

Previous research focused on cell-autonomous effects of GC and GR action within bone cells toward bone homeostasis and insights were provided by the use of cell type specific mutant mouse strains compromising GC signaling.

Intriguingly, GCs at the physiological levels have anabolic effects on bone. They promote the formation of osteoblasts from mesenchymal progenitor cells and are essential for maintaining bone homeostasis (19). This is evident from patients (20), since fracture risk is increased during adrenal insufficiency (21) and was shown experimentally through the use of mice that have either impaired GC metabolism in the osteoblast lineage or a selective deletion of the GR. Overexpression of the GC inactivating enzyme 11β-HSD2 in mice in early differentiated osteoblasts (22–24), but not at late differentiation stages (25) led to a reduction of cortical and trabecular bone mass in adult mice. Furthermore, a defective mineralization in the calvaria was observed which was associated with diminished Wnt Signaling (26). A reduced trabecular bone mass was also seen in mice lacking the GR in the osteoblast lineages using the Runx2 as a driver for the cre expression in the cre-loxP system (27). Furthermore, GR deficient cells displayed strongly diminished differentiation potential *in vitro*. Since osteocytes are also mutant in *GR*^Runx2Cre^ mice, currently it remains unclear how much the GR in osteocytes contributes to the bone mass at physiological conditions. Taken together, endogenous GC signaling via the GR promotes osteoblastogenesis. However, the GR is not essential for osteoblast generation. The embryonic lethal GR knockout mice (27) and mesenchymal specific GR knockout mice (28) displayed no absence of calcification in late stage embryos. Thus, GR is a positive modulator of osteoblastogenesis, but not a crucial factor. In contrast to the GR deletion in mesenchymal cells, deletion of GR in myeloid cells including macrophages, neutrophils, and osteoclasts, does not affect bone in adult mice in the absence of inflammation, indicating that osteo-immunological cross-talk in the absence of inflammation at physiological GC levels plays a minor role in controlling bone mass (27).

**Figure 1:**
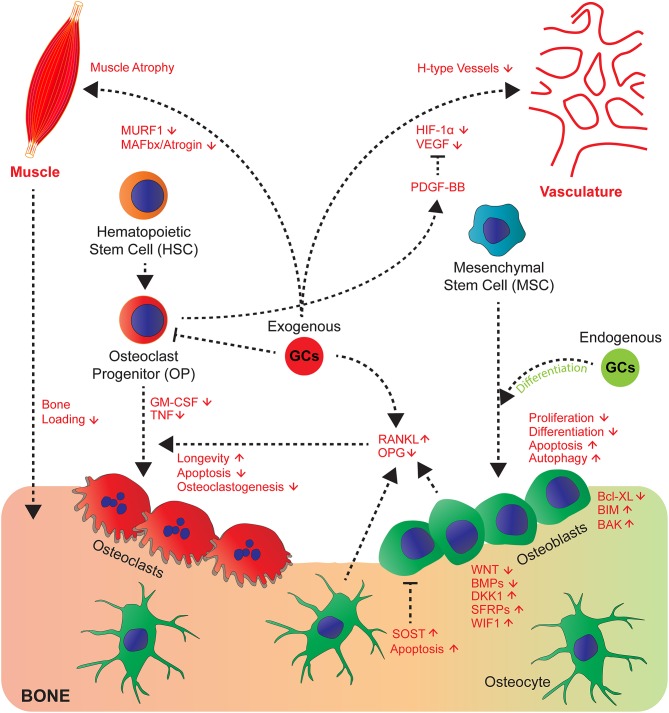
GCs affect cross-talk of bone cells and their communication with muscle, vasculature and myeloid cell-derived osteoclasts. GCs act directly and indirectly on bone, hematopoietic and mesenchymal cells and tissues that affect bone integrity. Endogenous GCs (green) rather favour differentiation of osteoblasts, whereas exogenous (red) rather decrease proliferation, differentiation and enhance apoptosis and autophagy of osteoblasts and osteocytes by differential regulation of signalling molecules of the Wnt and BMP pathway and pro- and anti-apoptotic molecules. Direct effects on osteoclasts are differential concerning longevity, apoptosis, osteoclastogenesis (for details see text) and indirect by altering RANKL/OPG ratio. GCs regulate cross-talk of vasculature toward bone and muscle toward bone by exerting modulatory effects on both systems (muscle atrophy) and likely impairing H-type vessels, since respective signalling molecules (VEGF and PDGF-BB are regulated by GCs).

This becomes strikingly altered at conditions with high exposure of GCs as it occurs in steroid therapy. GC-induced osteoporosis is among the most common so-called secondary osteoporosis (29), when bone loss is induced as side effects by medication. Here exogenous GCs have contrasting effects to endogenous GCs on osteoblasts, which decreases their proliferation (30), differentiation, and induce apoptosis and modulate autophagy (25, 31–35). Whereas, induction of autophagy seems not to be decisive for inhibition of osteoblast and osteocyte function *in vivo* (36), an impaired differentiation and induction of apoptosis likely lead to decreased bone formation rate (27, 33). The molecular mechanisms of the pharmacological effects on osteoblast function are partially understood. The inhibition of proliferation and differentiation is supposed to be due to inhibition of growth factors (IGF-1, WNT proteins, BMPs), expression and inhibiting the activity of their downstream signaling pathways [reviewed in (10, 19)]. The molecular mechanisms of this inhibition involves in part the induction of inhibitory molecules such as DKK1, Sclerostin, secreted frizzled and WIF1, all antagonizing Wnt signaling (19, 37). Furthermore, negative interference of the activity of the transcription factors AP-1 and Notch had been proposed (27, 38). Recently, the involvement of miRNAs was suggested (39, 40). This was challenged by a study showing that the abrogation of dicer dependent processing of miRNAs did not inhibit decreased bone formation by GCs in osteoblast specific mutant Dicer mice (41). The induction of osteoblast and osteocyte apoptosis, another cellular phenotype associated with decreased bone formation was attributed to suppression of the pro-survival gene Bcl-XL and increase of pro-apoptotic genes BIM and BAK (42–44). Additionally the generation of reactive oxygen species by rapid activation of pro-active kinases Pyk2, and JNK were suggested (45) (Figure 1).

GCs also directly act on osteoclasts stimulating initial resorption after high GC exposure (46), which then declines with prolonged GC exposures. These effects are known to be mediated through the stimulatory actions of GCs on proliferation and differentiation of osteoclast precursors as well as by prolongation of their longevity (47–49). In contrast, early progenitors are attenuated by GCs (48, 49). This latter effect might explain the decline of resorption at very long GC treatments. Nonetheless, once the osteoclasts had been formed GCs lead to enhanced longevity (46, 50), apoptosis could be suppressed, and the effects of receptor activator of nuclear factor kappa-B ligand (RANKL) potentiated. Importantly, this was abrogated in osteoclasts from GRA485T (*GR*^dim^) knock-in mice, with impaired GR dimerization (50, 51). This indicates that in contrast to GC-mediated suppression of bone formation for the increase of resorption, GR dimer dependent genetic programs are required.

### GCs Affecting Cellular Cross-Talk of Bone Cells

Since the observation that bone formation and bone resorption are functionally coupled at the bone remodeling unit (52), cross-talk of cells in bone was considered as a hall mark of bone metabolism. This observation was supported by the discovery that osteoblasts and osteocytes are regulating bone resorption by triggering osteoclastogenesis via the induction of the pro-osteoclastogenic factor RANKL (53, 54) following exposure to M-CSF. This occurs in response to systemic hormones, such as PTH. RANKL on the other hand is counteracted by OPG. GCs seem to affect this cross-talk in part as well, since GCs induce RANKL and suppress OPG in osteoblastic cells, affecting bone resorption (27, 55–57) (Figure 1). RANKL inhibition by Denosumab in humanized mice improved some, but not all parameters of bone loss to GC effects (57). For the increase of osteoclasts in cortical bone, RANKL expression in osteocytes is decisive as shown by Piemontese et al. using mice with a conditional deletion of RANKL using *Rankl*^Dmp1Cre^ mice (58).

Besides, the crucial soluble factors RANKL and M-CSF, TNF and TREM2 ligands play a decisive role in commitment, fusion and maturation of osteoclasts (59). Of these, TNF expression is strongly reduced by GCs at a transcriptional and post-transcriptional level. GM-CSF itself is reduced by the GR via interaction with NF-AT/AP-1 binding sites in the enhancer of the GM-CSF gene (60) (Figure 1). This is consistent with the observation that the onset of osteoclastogenesis is inhibited by GCs, which depends on cell autonomous effects (48, 49) and the down regulation of extracellular mediators. The latter was shown by coculture experiments where GCs strongly suppressed osteoclastogenesis dependent on the GR in osteoblasts despite the GR deficiency in osteoclast progenitors (27). This might play in particular a role during inflammation, where osteoclastogenesis and resorption is usually enhanced, and might be beneficially counteracted by GCs. Whether other osteoclast regulatory extracellular factors are under the control of GCs and whether this matters for osteoclastogenesis and activity is still unexplored.

Even less is understood, whether GCs affect osteoclast signals toward osteoblasts or osteocytes. This is still due to the paucity of knowledge of osteoclast-derived factors influencing osteoblasts and osteocytes. Among these identified are ephrinB2, the D2 isoforms of vacuolar (H+) ATPase (v-ATPase) V0 domain (Atp6v0d2), the complement component 3a, semaphorin 4D and microRNAs [reviewed in (61)]. It is not known whether any of these are regulated by GCs to our knowledge. Regulation of microRNAs had been shown for cell-autonomous effects in osteoblasts and osteoclasts, respectively, but whether the osteoclast-osteoblast communication or *vice versa* is affected is unknown. Thus, for this type of cross talk there is tremendous scope for research.

### GCs Influencing Cross-Talk of Vasculature and Bone Cells

Bone is highly vascularized and previous work demonstrated that vascularization and angiogenesis is coupled with bone growth and bone homeostasis (62–64). GCs have a profound inhibitory action on vasculogenesis in bone accompanied by inhibition of HIF-1α and its target gene vascular endothelial growth factor (VEGF) (65). This is accompanied by edema formation in the femoral head in mouse bone, an area with considerable amount of vessel remodeling. In OG2-11β-HSD2 transgenic mice, overexpressing the GC inactivating enzyme 11β-HSD2 in osteocalcin expressing cells, the decrease of vasculature volume was in part prevented (62, 65). Recent studies identified the presence of a subtype of vessels, so-called H-Type vessels, positive for CD31 and endocmucin being associated with bone formation (63). These H-Type vessels were found to be reduced by GC excess, a process that could be prevented by addition of platelet-derived growth factor-BB (PDGF-BB) (66). Since PDGF-BB is in part derived from osteoclast progenitors (67), PDGF-BB could be a factor targeted by GCs.

Taken together, the precise contribution of GC signaling in cells of the vasculature vs. osteoclasts, osteoblasts and osteocytes remain to be determined, which will be of importance to decipher the effects of GC excess on bone integrity.

### GCs Influencing Cross-Talk of Muscle and Bone

Since GC excess does not only influence bone strength, but also leads to muscle atrophy, this increases the risks of falls and reduces load on bone, thus accelerating bone loss and increasing fracture risk (68). GCs induce protein degradation in muscles associated with induced FoxO-dependent expression of E3 ubiquitin ligases atrophy F-Box [MAFbx/atrogin and muscle RING finger 1 (MURF1)], which is mediated in part through the GR in muscle (69–71). Surprisingly, some of these genes are also regulated in bone by excessive GC amounts (68), suggesting that some deleterious pathways might be shared between bone and muscle. The cross-talk between muscle and bone exist beyond the mechanical load. Kim et al., discovered that the muscle derived hormone Irisin binds to alphaV class of integrins in osteocytes and might stimulate resorption and increased sclerostin expression (72). Whether further soluble factors participate in this muscle bone cross-talk and whether they or Irisin signaling itself, are a target of GCs remains to be investigated. Nonetheless, both direct effects on muscle and on bone cells accelerate weakness of bone.

Interestingly, in the absence of inflammation, models of GC induced osteoporosis so far provide no clear evidence of regulation of the cross-talk between bone cells such as osteoblast/osteocytes with innate immune cells, except osteoclasts and their progenitors. This does not mean that GC mediated regulation of this cross-talk does not exist. However, this has not been addressed so far with appropriate cell conditional mouse models. This is completely different for conditions of inflammation in bone described below, where regulation of cross-talk emerges as a major theme for limiting inflammation at least in arthritis.

## GC Effects on Inflammatory Bone Diseases—Direct Effects and Effects on Stromal-immune Cell Cross-talk

### Effects of GCs on Innate Immune Cells

Innate immune cells, in particular mast cells, tissue macrophages, neutrophils and other cell types secrete inflammatory mediators (cytokines and vasodilator agents) during chronic inflammation, as it occurs e.g., during tissue damage. GCs are known to suppress the production of inflammatory mediators partially by acting on Toll-like receptor (TLR) signaling (73, 74). They also act on macrophages to inhibit the production of eicosanoids, which are lipid mediators that promote vascular dilation and permeability (75, 76). GCs also reduce the blood flow to inflammatory sites by sensitizing endothelial cells to vasoconstrictors and by inhibiting the production of vasodilators (77). In addition, GCs attenuate leukocyte extravasation by inhibiting transcription of integrins and their ligands, intercellular adhesion molecule 1 (ICAM1) as an example (78, 79). Finally, GCs inhibit the expression of many pro-inflammatory cytokines and chemokines. Mice with conditional GR ablation in macrophages or dendritic cells (DCs), produced higher levels of IL-1β, IL-6, TNF, and IL-12, and exhibited greater mortality during experimentally induced sepsis (80–82). Whereas, downregulation of chemokines, such as CC-chemokine ligand 2 (CCL2), CCL3, CCL5, restrains leukocyte migration, and deficiency of macrophage-recruiting molecule MCP-1 in mice (also known as C-C motif chemokine receptor 2 [CCR-2]), led to compromised fracture healing (83).

Interestingly, GCs reduce mast cell number, maturation and activation (84–87) and stabilize mast cells dose-dependently by inhibiting their exocytotic process. This effect is ascribed to the non-genomic actions of GCs, acting via the GR present in the plasma membrane of mast cells, and directly influencing the intracellular Ca^2+^ signaling pathway (88). In a mouse model of 11β-HSD1 deficiency, reduced intracellular GC action in mast cells correlated with increased activation demonstrating a clear influence of 11β-HSD1 on mast cell degranulation (89).

Despite suppressing inflammatory activity of immune cells, the concept emerges that GCs terminate inflammation by polarizing cells toward an anti-inflammatory phenotype. This has been thoroughly investigated in macrophages. Several studies demonstrated that GCs induce specific differentiation of monocytes with an anti-inflammatory phenotype and promote their survival, contributing majorly to the resolution of inflammation (90–93).

The induction of anti-inflammatory acting immune cells is decisive for resolution of inflammation during fracture healing and arthritis and is subject to GC action.

## Glucocorticoids (GCs) and Fracture Healing

### Cells Involved in Fracture Healing

The role of GCs during fracture healing, a process that requires multiple communication steps between different cell types, is not well-understood. Fracture healing involves close interaction between bone cells and immune cells. Bone injury causes the onset of inflammation. A fracture hematoma is formed containing DAMPs and PAMPs (danger/pathogen-associated molecular patterns), erythrocytes, inflammatory cytokines and cells of the innate immunity. The inflammatory phase is followed by the repair phase where a cartilaginous callus is formed and then remodeled by osteoblast and osteoclasts (94).

**Figure 2:**
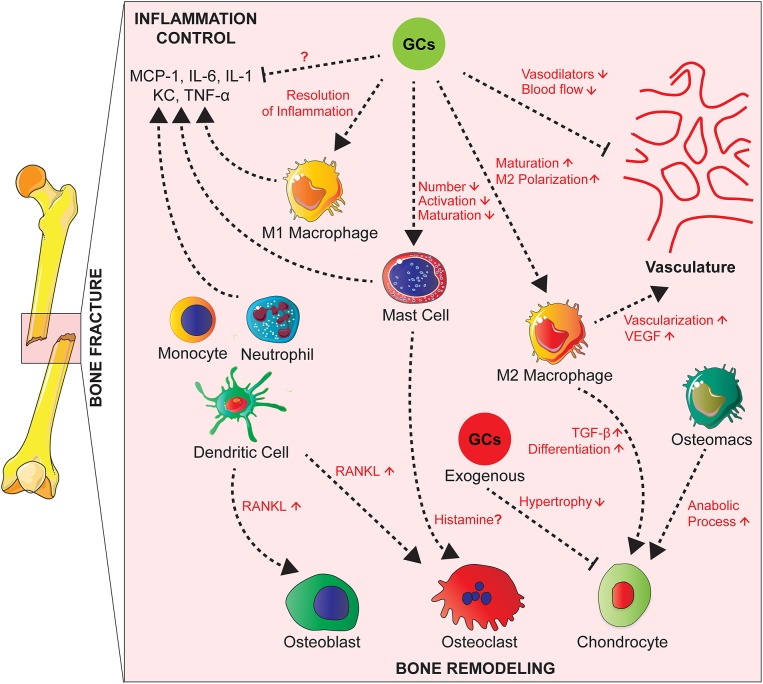
Effects of GCs on the cross-talk between cells of the innate immunity, bone cells, and vascularization during fracture healing. During fracture healing, cells of the innate immunity such as neutrophils, macrophages and mast cells produce pro-inflammatory cytokines and attract other phagocytes to remove debris. GCs act on these cell types to control the inflammation and resolve it partially by polarizing macrophages toward an anti-inflammatory phenotype that will in turn promote tissue repair by increasing vascularization. Presumably GCs also inhibit M1 macrophages and cytokine expression, which is not proven yet. On the other hand, GCs have counterbalancing effects by inhibiting the production of vasodilators in order to control the inflammation. Innate immune cells act on bone cells by secreting factors such as RANKL affecting then osteoblast and osteoclast activity. Also, tissue resident and infiltrating macrophages play a role in endochondral ossification by promoting chondrocyte differentiation for callus formation. Chronic GCs treatment delays chondrocyte hypertrophy and attenuates endochondral bone healing.

Several innate immune cells are present in the early fracture hematoma such as neutrophils, macrophages and mast cells (95–98). Activated mast cells release inflammatory mediators, including histamine, KC, IL-1β, TNF, and IL-6, as well as various chemokines attracting other immune cells (99, 100). Neutrophils and macrophages migrate to the injury site in response to inflammatory mediators to phagocytose debris and pathogens (96, 97, 101, 102) (Figure 2).

Depletion of neutrophils, leads to impairment of fracture healing in mice (95, 103), and a reduction of mesenchymal tissue repair in a rat model of growth plate injury (104). Macrophages persist during all phases of fracture repair (96, 97), where they are important for bone healing (105). In a mouse model of femoral fracture, Raggatt et al. showed that inflammatory macrophages were required for the initiation of the fracture repair, while both inflammatory and osteomacs, specialized resident bone macrophages, promoted anabolic processes during endochondral callus formation (106). Mast cell deficiency in mice, however, causes a reduction of the inflammatory response after fracture and disturbed callus remodeling. In the same study, *in vitro* investigation suggested histamine as a major mediator of mast cells action on osteoclastogenesis (98) (Figure 2).

During the repair phase, mesenchymal precursors, close to the site of the fracture, differentiate into chondrocytes and start the process of endochondral ossification. A cartilaginous soft callus is formed in order to stabilize the fracture (107). Under stable mechanical conditions the vascularization of the callus is initiated and subsequently followed by its mineralization and its conversion into bone (108). Finally, the callus is remodeled by osteoclasts and osteoblasts and the original bone architecture is restored (109).

Cells of the immune system influence the process of endochondral ossification. Tissue resident and infiltrating macrophages, in particular M2 macrophages enhance vascularization by secreting VEGF at the fracture site (97). They also release TGF-β that plays a pivotal role in chondrogenic differentiation of mesenchymal stem cells for callus formation (110). Monocytes, neutrophils, DC, and B and T lymphocytes produce RANKL and subsequently influence osteoclast and osteoblast activity (111, 112) (Figure 2).

### Effects of GCs on Fracture Healing

The injury represents a stress stimulus that triggers endogenous GC release to control the inflammation. We have previously shown, in a mouse model of fracture, that mice with an induced global deletion of the GR, including bone and immune cells, had an impaired fracture healing. The presence of the GR had a protective role in our model partially by shaping the inflammatory response (113).

Few studies investigated the effects of synthetic GCs on fracture healing. It was shown that short-term treatment with GCs had minor effects on bone repair (114) while long-term treatments significantly impaired the healing process (115, 116). In a medaka fish fracture model, although both chronic and acute GC treatment affected osteoclast recruitment and osteoblast accumulation, only chronic GC treatment significantly delayed the healing (117).

The role of GCs on endochondral ossification in fracture healing hasn't been widely investigated. In a model of glucocorticoid-induced osteoporosis, endochondral ossification was impaired after fracture as chondrocyte hypertrophy was delayed (118). In a tibial metaphyseal fracture model, GR deletion in chondrocytes attenuated endochondral bone healing by momentarily increasing the cartilage content of the callus, but didn't impact negatively on the healing outcome (119). In contrast, treatment with dexamethasone had an inhibitory effect on healing in the femur shaft fracture in comparison to metaphyseal fracture, suggesting a more important role of GCs in endochondral rather than intramembranous ossification (120).

Given the distinct roles of GCs on cross-talk of immune, bone and stromal cells, and on vasculature and muscle during osteoporosis and arthritis, it is very likely that GCs shape different aspects of fracture healing positively and negatively. The exact interplay requires intensive investigations.

## Glucocorticoids (GCs) in Osteoarthritis

### GC Effects on Osteoarthritis

Osteoarthritis (OA) is the most common form of arthritis and the leading cause of pain and disability in elder people (121). The clinical picture includes not only a process of “wear and tear” but also an unbalanced remodeling of the joint associated with inflammatory processes (122). Among the main risk factors for OA are obesity, gender and age (123).

Degeneration of joints occurs as damage in articular cartilage and subchondral bone, accompanied by ectopic bone formation, so-called osteophytes. The slow turnover of extracellular matrix is dramatically enhanced in OA due to secretion of degrading proteinases and consequent loss of proteoglycans and collagen (124). This process is likely triggered by a vicious cycle of cross-talk of inflammatory cells and stromal cells, such as chondrocytes and synovial cells.

**Figure 3:**
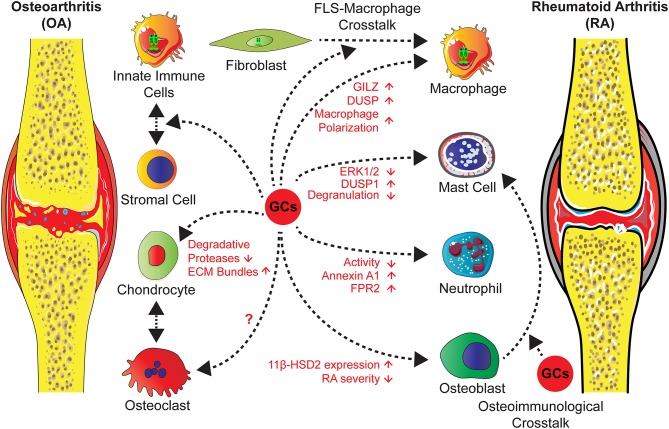
GCs administration in the treatment of OA and RA exert direct effects on different cell types and influence stromal-immune cell crosstalk. Actions of GCs on neutrophils and mast cells lead to an attenuated inflammation and an induction of anti-inflammatory mediators. GCs operate on macrophages either directly, causing increased levels of GILZ and decreased inflammation, or indirectly via FLS through a cross-talk between both, leading to a shift of macrophage polarization toward an anti-inflammatory phenotype and an increased efferocytosis activity. Further effects on stromal cells, in particular chondrocytes are a reduction of degradative protease levels and an increase of ECM molecules. Concerning cross-talk between osteoclasts and chondrocytes in OA or in RA the influence of GCs are unknown. Strikingly, in RA osteoblasts GC effects might lead to inflammatory and erosive processes, since the overexpression of the GC inactivating enzyme 11β-HSD2 in osteoblasts, results in an attenuated disease severity by a non defined cross-talk.

The role of endogenous GCs in this process is obscure, a recent study of Tu et al., however, showed that overexpression of the GC inactivating enzyme 11β-HSD2 in osteoblasts in transgenic mice attenuates OA in a model of destabilization of the medial meniscus (DMM) in older mice (125). This indicates that in bone cells GCs might trigger the inflammatory and erosive process (Figure 3).

Administered GCs are accepted as short term, but not as long term agents for intra-articular injections of knee OA with few side effects [reviewed in (10)]. In the literature, the mechanisms are attributed to effects on stromal cells, by increasing the expression of ECM molecules and reduction of degradative proteases. This had been investigated in OA models, cartilage explant cultures and in cartilage cell lines [reviewed in (10, 126)] (Figure 3).

Macrophages are part of the inflammatory infiltrate in OA (127). Interestingly, a depletion of synovial macrophages led to the augmentation of OA in a model of destabilization of the medial meniscus (DMM) combined with high fat diet (128). The absence of macrophages caused intriguingly an increased numbers of T cells indicated a hyperinflammation. This indicates that anti-inflammatory polarized macrophages are essential to prevent aberrant progression of OA.

The precise contribution of GR in immune cells for GC effects on OA has not been addressed mechanistically so far. Furthermore, the suppression of VEGF by GCs (129), strongly suggests that effects on the vasculature, could be beneficial to facilitate repair processes during acute GC exposure. Long term effects on the vasculature could also be non-favorably and trigger further OA progression.

Overall, the GC action in OA is not completely understood and requires further elucidation given that GCs are frequently used for treatment, and that obesity, stress and age are known risk factors for the development of this pathology.

### GC Effects on Osteophytes in Arthritis

Beside the effects of GCs on joint erosion, not much is known about their protective effects against ectopically grown bone, so-called osteophytes. In both, inflammatory and osteoarthritis, osteophytes can be observed (130, 131) and result in pain and loss of function of joints (132). Osteophytes arise from periosteal mesenchymal stem cells (MSCs) that undergo chondrogenic differentiation, mature and produce a cartilaginous scaffold that is replaced by bone in the end-stage of osteophyte formation (133), a process closely related to endochondral ossification (130, 134–137). GCs are shown to suppress osteophyte formation (138–140), as well as endochondral ossification (141), however, it is uncertain whether the same mechanisms are involved. Interestingly, besides MSCs and chondrocytes, cross-talk with components of the innate immunity are shown to play an important role in the initiation of osteophytes (142–144). Osteophytes often develop in close proximity to synovial lining (144) and synovial inflammation is considered a key contributor to osteophyte formation (145). In this regard, it was shown that a single low-dose of avidin-conjugated dexamethasone (Dex) suppress synovial infiltration and osteophyte formation in post-traumatic OA (138). Especially, synovial macrophages, as part of the synovial infiltrate, are considered key players in osteophyte formation as their depletion significantly suppress osteophytes in two different mouse models of OA (142, 144) and GC-mediated inhibition of synovial macrophages might be beneficial to prevent osteophyte formation. Interestingly, inhibition of TNF does not result in the reduction of osteophytes in patients with psoriatic arthritis or mouse models of inflammatory arthritis (146, 147). Thus, GC-mediated suppression of pro-inflammatory cytokines alone might not be sufficient to suppress osteophyte formation. On the other hand, damage-associated molecules derived from degrading cartilage (142) can also activate synovial macrophages and depending on dosage and duration, GC treatment can protect against this cartilage degradation in OA (140, 148) and inflammatory arthritis (149). In this regard, experimental reduction of cartilage degradation reduces formation of osteophytes in mouse models of OA (150, 151).

The most prominent pathways involved in synovial macrophage activation and osteophyte growth are transforming growth factor β (TGFβ) and bone morphogenetic proteins (BMP-2/-4) (142, 144, 152, 153). TGFβ and BMPs initiate chondrogenic differentiation from periosteal MSCs and co-cultures of MSCs and macrophages enhanced spheroid formation after TGFβ treatment when compared to MSCs alone (144). Interestingly, macrophage-specific delivery of liposomal packed prednisolone results in down regulation of TGFβ in inflammatory arthritis (154) and Dex treatment was shown to suppress BMP-signaling and induce BMP-antagonists at least in osteoblast cell lines (155, 156). In addition, blockage of the hedgehog-signaling pathway also resulted in the suppression of TGFβ and BMPs and completely prevented osteophyte formation without affecting synovial inflammation (157). Thereby, GC-mediated control of TGFβ and BMPs might counteract osteophyte formation. Surprisingly, intra-articular injections of triamcinonone acetonide (TA, another GC) were associated with a higher macrophage activity, using folate-based radiotracers, but also resulted in a significant reduction of osteophytes (158). *In vitro* results of Siebelt et al. (158) suggested that the induction of CD163, folate receptor-β and interleukin-10 by TA might play a role in osteophyte suppression (158), however, this needs to be validated *in vivo*. Besides TGFβ and BMPs, dickkopf-1 (Dkk1), a master regulator of bone remodeling is strongly regulated by GCs (159) and is involved in osteophyte formation (160). Inhibition of Dkk1 results in osteophyte formation in an inflammatory mouse model that does not initially develop osteophytes (160). In addition, patients with spondylarthritis (SpA) arthritis that do develop osteophytes, show lower levels of Dkk1 (161), whereas rheumatoid arthritis (RA) patients that do not develop osteophytes have higher levels of Dkk1 (162). GCs, however, strongly induce Dkk1 expression and thereby inhibiting osteoblast differentiation and bone formation (163, 164), which might be beneficial to suppress osteophyte growth. Accordingly, overexpression of Dkk1 in the osteoblast-lineage significantly reduces osteophyte size in OA (165).

In contrast to exogenous GCs, disruption of endogenous GC signaling in the osteoblast-lineage reduces osteophyte formation in an age-related OA mouse model suggesting an osteophyte-promoting role of endogenous GC (125). Further experimental work is needed to discriminate the endogenous vs. the exogenous effects of GCs on osteophyte formation and to validate potential pathways involved in GC-mediated suppression of osteophytes to better understand the crosstalk of bone and immune cells involved in this process.

## Rheumatoid Arthritis (RA)

Rheumatoid arthritis (RA) is a chronic, autoimmune disease with a worldwide prevalence of 0.5–1% (166). It affects all types of patients with the highest occurrence in elderly women and a female to male ratio of 3:1 (167). RA is associated with several contributing factors, such as genetics, smoking, obesity and the environment (168). A hallmark of RA is synovial inflammation and the destruction of cartilage and bone, which makes RA a bona-fide disease of osteo-immunological interactions. The etiology is still to a certain extend unclear, but involves rheumatoid factor and anti-citrullinated peptide antibodies (ACPAs), which are at least predictive for the development of RA. The expression of pro-inflammatory mediators, like TNF and IL-6 activating the innate immune system concomitant with aberrant T- and B-Cell regulation finally leads to the development of autoantibodies (169). In the joints, osteoclasts activated by citrullinated autoantibodies, lead to bone damage. This further results in cytokine release by local cells and activation of synovial fibroblasts and macrophages (170, 171), exaggerating the inflammatory and destructive response.

Since the discovery of their anti-inflammatory action 70 years ago, GCs are still one of the most frequently used medications to treat the acute inflammatory response in RA.

Our knowledge of the mechanisms of action of GCs rely on different animal models that comply with certain aspects of the inflammatory phase in arthritis, such as collagen-induced arthritis (involving aspects of T-cells, mast cells and macrophage functions), antigen-induced arthritis (strictly T cell dependent), serum transfer-induced arthritis (T-cell independent) and TNFα transgenic mice (involving multiple cell types).

In the serum transfer-induced arthritis (STIA) and TNF-transgenic model of arthritis, it could be shown that a deficiency of 11β-HSD1 leads to an increase of inflammation, suggesting attenuation of endogenous GC action (172, 173). However, in another model of collagen-induced arthritis (CIA), 11β-HSD1 deletion caused an attenuation of inflammation indicating a pro-inflammatory role of GC activation in this model.

A clear anti-inflammatory role for the GR could be demonstrated in these models (Figure 3). For this, the capacity of the GR for dimerization seems to be required for suppression of inflammation in all arthritis models tested so far. GRA458T (*GR*^dim^) knock-in mice with attenuated GR dimerization (51), but intact monomer activity, were found refractory in arthritis models of antigen-induced arthritis, glucose-6 phosphate isomerase (G6PI)-induced arthritis and STIA (174, 175). Thus, GR dimerization-induced gene regulation seems to be a general mechanism and is in accordance with animal models with disturbed GR dimer-dependent target genes of the GR such as mitogen-activated protein kinase phosphatase 1 (MKP1), Glucocorticoid-induced leucine zipper (GILZ), and Annexin A1 (Figure 3).

GILZ interacts with several crucial signaling pathways, such as NF-κB signaling and T-cell activation (176). GILZ is constantly produced in macrophages and is stimulated by GCs and IL-10, thereby mediating the deactivation of macrophages and thus a decrease of macrophage infiltration (177). This regulation affects the balance between intensified immune reactions and immune tolerance. In mice with CIA and in human patients with RA, it could be shown that GILZ was upregulated in the synovium after the administration of GCs. Furthermore, in cultured RA synovial fibroblasts, an overexpression of GILZ inhibited the release of IL-6 and IL-8 (178).

DUSP1/MKP1 is induced by the GR dimer (179), and an important mediator of anti-inflammatory actions of the GR (81, 180, 181). It inhibits MAP Kinase signaling and DUSP-1 knockout mice have an earlier onset and higher score in CIA (182).

Annexin A1 is associated with the adaptive and the innate immunity. The anti-inflammatory effects of GCs are partly regulated by the release of Annexin A1 and the activation of its receptor formyl peptide receptor 2 (FPR2, also known as ALXR) in neutrophils and macrophages (183). Annexin A1 deficient animals render resistant to GCs in STIA (184), indicating a pivotal role for inhibition of inflammation.

### Cell Type Specific GC Action and Crosstalk Between Immune— and Stromal Cells

Depending on the model used different cell type specific requirements for GC signaling and the GR were suggested to attenuate arthritis. For the T-cell dependent antigen-induced arthritis indeed the GR in T cells is absolutely essential for GC-mediated immune suppression in part by suppressing the generation of IL-17 producing T-cells (174). In contrast in the STIA model the deletion of GR in T-cells does not attenuate the response toward GCs (175). Strikingly, in both models GR deletion in macrophages in *GR*^LysMCre^ mice hardly affected the efficiency of suppression of inflammation (174, 175). This is surprising, since there is multiple evidence for macrophages to respond to GCs during inflammation in general and the requirement of the GR in models of systemic inflammation, contact allergy and acute lung injury (80, 81, 185). In addition in STIA the presence of alternative activating macrophages is decisive for resolution of inflammation (186).

Despite other immune cells, such as type 2 innate lymphoid (ILC2) cells or others, have not been exploited yet for their functional relevance of anti-inflammatory efficacy, a new theme is emerging demonstrating the role of GR in non-immune cells.

Genetic inhibition of GC signaling in osteoblasts by overexpression of 11β-HSD2 lead surprisingly to an attenuated STIA (187). The mechanism is not clear yet, but maybe in accordance to the global 11β-HSD1 deletion in CIA.

In contrast, deletion of GR in chondrocytes in GR Col2a1CreER^T2^ mice leads to an accelerated inflammation in both CIA and STIA model (188). This was accompanied by an increased CXCR2 expression in the joint suggesting that GR controls chondrocyte-immune cell cross-talk on the level of CXL2/5 CXCR2 chemokine axis involved in leukocyte recruitment.

A recent study showed that GC actions in stromal cells are decisive and GR expression in immune cells alone is not sufficient to suppress inflammation in STIA (175) (Figure 3). Experiments in bone marrow chimeric mice lacking the GR in the hematopoietic compartment showed no differences in the onset or progression of STIA, nor the responsiveness to GC treatment compared to chimeric mice with a functional GR in immune cells. Furthermore, a reverse approach with chimeric mice lacking the GR globally except for the hematopoietic system revealed that GR expression in stromal cells is essential for the anti-inflammatory actions of GCs. More precisely, the study showed that for these anti-inflammatory actions, the homodimer form of the GR in stromal cells is critical. Interestingly, deficiency of GR dimerization in these cells had no effect on the suppression of inflammatory cytokines upon GC treatment. This indicates that their decrease alone is not sufficient to suppress inflammation. Additionally, GR dimers in stromal cells induce non-classical, anti-inflammatory macrophages while the levels of classical macrophages are not altered. Several anti-inflammatory markers, associated with enhanced phagocytosis and efferocytosis activity, are increased only in wildtype (wt) but not in GR dimer deficient stromal cells. This suggests an insufficient clearance of apoptotic cells after GC treatment, which leads to a persisting inflammatory condition. Finally, the study suggests that the induction of anti-inflammatory macrophages may be indirectly guided by actions of stromal cells, in particular fibroblast-like synoviocytes (FLS), since cocultures of macrophages and FLS showed an elevated efferocytosis competence when compared to cocultures of macrophages and GR dimer-deficient FLS. In addition to that, the levels of macrophage associated chemokines macrophage inflammatory protein−1α and−1β (Mip-1α / Mip-1β) are decreased in wt but not in GR dimer-deficient FLS after GC treatment. Taken together, this indicates a GC-mediated, GR dimer-dependent cross-talk between FLS and macrophages that induces an increase in the anti-inflammatory macrophage population and thereby a suppression of inflammation and STIA itself (175) (Figure 3).

## Overall Conclusion/Outlook

Overall GCs and the GR have complex actions in bone diseases. The power of conditional mouse genetics demonstrated that GC signaling and GR action in distinct cell types of the immune system, stromal cells and bone cells have different contributions to the overall effects of GCs. Moreover, going away from this simplistic approach of interpreting cell type specific—cell autonomous effects, the field is now moving toward understanding the impact of GCs on interactions of distinct cell types or even organs.

Other issues that remain unexplored are the interplay of GC triggered immune cells in the normal pathology of postmenopausal and age-related osteoporosis. This is striking since the immune cells from the bone marrow need the bone as a niche, therefore strong interactions of immune and bone cells occur as a normal physiological process.

Given that GCs are part of the neuroendocrine regulatory network that also control inflammation and healthy bone homeostasis, a more holistic view will be needed. With the technologies of high content analysis, single cell sequencing and systemic approaches in combination with organoid models and carefully interpreted animal models, our understanding will substantially increase about the influence of these versatile hormones on the immune-metabolic crosstalk.

## Author Contributions

MA, YH, FM, KP, MK, and JT wrote individual sections of the article. MA, YH, and KP generated the figures.

### Conflict of Interest

The authors declare that the research was conducted in the absence of any commercial or financial relationships that could be construed as a potential conflict of interest.
